# Comprehensive Review of Coronary Artery Anatomy Relevant to Cardiac Surgery

**DOI:** 10.2174/011573403X321942241023112517

**Published:** 2024-10-31

**Authors:** Emeka B. Kesieme, Benjamin Omoregbee, Dumbor L. Ngaage, Mark H.D. Danton

**Affiliations:** 1 Department of Cardiothoracic Surgery, Castle Hill Hospital, Cottingham, United Kingdom;; 2 Adult Cardiac Surgery, Castle Hill Hospital, Cottingham, United Kingdom;; 3 Congenital Cardiac Surgery, Royal Hospital for Children, Glasgow, United Kingdom

**Keywords:** Right coronary artery, left coronary artery, coronary artery variants, dominant circulation, coronary collaterals, coronary anatomy

## Abstract

In order to perform safe cardiac surgery, a knowledge of applied coronary artery anatomy and its variants is essential for cardiac surgeons. In normal individuals, the right and the left coronary arteries arise from the corresponding sinuses of Valsalva within the aortic root. From the cardiac surgical perspective, the coronary artery is divided into the left main coronary artery, its branches (the left anterior descending artery and the circumflex artery), and the right coronary artery. With high-risk cardiac surgeries, including redo procedures, becoming increasingly performed, abnormal courses and variations of the coronary arteries, if not recognized, can predispose the patient to avoidable coronary injuries, resulting in adverse outcomes of cardiac surgical procedures. We aim to describe normal and applied coronary anatomy, common coronary artery variants previously reported, and their clinical relevance to both adult and paediatric cardiac surgery.

## 1 INTRODUCTION

The right coronary artery (RCA) originates from the right coronary sinus, while the left main coronary artery (LMCA) originates from the left sinus of Valsalva and bifurcate to give rise to the circumflex artery and left anterior descending artery (LAD) [1].

The blood supply of the conduction pathway is mainly from the RCA. The branch to the sinoatrial (SA) node originates proximally from the RCA, while the branch to the atrioventricular node originates distally from the dominant RCA [2].

The vessel that gives rise to the posterior descending artery (PDA) (which supplies the posterior part of the interventricular septum), and also provides a branch to the atrioventricular node is the dominant circulation [3]. In right dominant circulation, the RCA gives off the PDA. In left dominant circulation, the circumflex gives off the PDA, and in codominant circulation, the PDA comes off both the RCA and the circumflex artery. About 70-80% of individuals have a right dominant circulation, while 5-10% have a left dominant circulation, and 10-20% have a codominant circulation [4].

Abnormalities involving the coronary arteries may be classified as anomalous origin, ectopic origin, solitary coronary artery, abnormal distal connections, and miscellaneous anomalies of the intrinsic anatomy [5].

This is a comprehensive review of the applied coronary artery anatomy and the common variations of the coronary artery anatomy that have been previously reported. The clinical relevance to both adult and paediatric cardiac surgery has also been extensively discussed.

## 2 METHODS

A literature review of coronary artery anatomy was done from 1940 to date using a manual library search, and journal publications on PubMed/Medline, Google Scholar, and EMBASE.

We used the following keywords: coronary artery anatomy/left main coronary artery/left anterior descending artery/circumflex artery/right coronary artery/posterior descending artery/branches/injuries/congenital abnormalities/.

Full texts of the materials, including those of relevant references, were collected and studied. Information relating to gross and applied anatomy of the coronary artery and its branches, congenital abnormalities involving the coronary artery and its branches, injury to the coronary artery in adults, and congenital cardiac surgeries were extracted from original articles, review articles, and relevant case reports.

## 3 DISCUSSION

### 3.1 Applied Anatomy of the Right Coronary Artery (RCA)

**Fig. (1):**
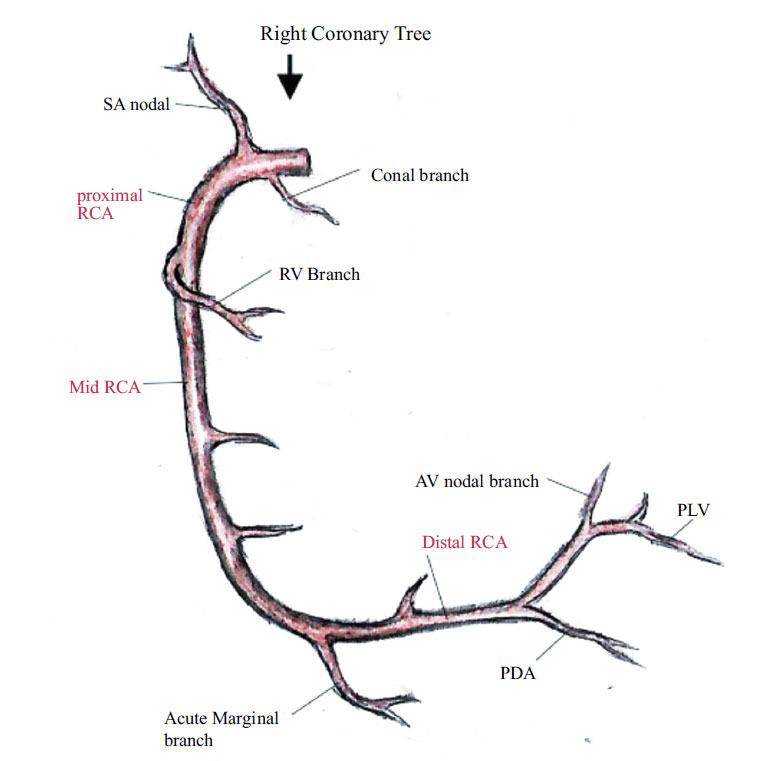
RCA and its branches.

This artery originates from the right sinus of Valsalva. It descends into the fat pad of the atrioventricular groove between the right atrium and the right ventricle towards the crux of the heart. It gives off branches as it courses down the sulcus (Fig. **1**) [5].

### 3.2 Infundibular or Conal Branch

The infundibular or conal branch is the first branch of the RCA. It travels anteriorly to the cephalad part of the right ventricle to supply the infundibulum of the right ventricle [6]. The conal branch is called the ‘third’ coronary artery when it originates directly from the right aortic sinus [7].

The conal branch from the RCA may communicate with the infundibular (conal) branch of the LAD when present to form an anastomosis called Vieussens’ ring or arterial circle of Vieussens. This ring can provide important collateral flow to an occluded LAD, thereby reducing ischaemia and preserving ventricular function despite the significant obstruction [8-10]. It also provides important collateral to the unrepaired anomalous left coronary artery from the pulmonary artery (ALCAPA) or failed ALCAPA repair.

### 3.3 Artery to the Sinoatrial (SA) Node

The branch to the sinoatrial (SA) node from the RCA is present in 60-70% of people. In the rest of the population, it arises from the circumflex artery [11]. Dual blood supply to the SA node from both RCA and circumflex is rare but possible [11]. In addition to the SA node, this artery also supplies blood to the Bachmann’s bundle, a bundle of parallel myocardial strands connecting the right and left atrial walls, cristae terminalis, and the free walls of the right atrium [12, 13]. After its origin, it runs along the anterior interatrial groove towards the superior cavoatrial junction [14]. Variations of origin and course of this artery may be seen in patients with complete transposition [15]. In this group of patients, the branch to the SA node may arise from the lateral branch of RCA and runs a higher risk of injury during the atrial incision utilized in the Mustard procedure [15]. When it runs deep in the interatrial wall beneath the interatrial band, it can be caught by sutures anchoring the baffle in the Mustard procedure [15].

The relationship of SA nodal artery to the superior vena cava (SVC) may be precaval, retrocaval, or pericaval. They may also present as an S-shaped subtype, which is characteristically rare, has a longer course, is larger than the other subtypes and supplies a larger area of the atrium than observed with the normal SA nodal artery [11, 13, 16].

Iatrogenic injury to the SA nodal artery can occur during open heart surgeries performed through the right atrium if the incision is made close to the cavoatrial junction [16]. The incision required to create an intra-SVC baffle in patients with sinus venosus atrial septal defect (ASD) may inadvertently injure the SA nodal artery. This is particularly more common in retrocaval or pericaval subtypes [17].

In the Warden repair of Partial Anomalous Pulmonary Venous Connection (PAPVC), a congenital malformation characterized by the drainage of one or more pulmonary veins (PVs) into the systemic venous circulation, one of the key principles is to avoid incision across the SA nodal artery and in the vicinity of the sinus node [18, 19].

Damage to the artery can also occur while utilizing the superior transseptal approach for mitral valve surgeries [20]. This complication is observed most especially among patients with the precaval subtype [12, 20].

Injury may occur during surgical ablation like Cox-Maze surgery, and this is especially more common in patients with the S-shaped course SA nodal artery [12, 16, 20].

### 3.4 Artery to the Atrioventricular (AV) Node

The branch to the Atrioventricular (AV) node arises from the U-shaped segment of the RCA at the level of the crux cordis in 90% of the population and in those with right or balanced dominance [21]. This artery comes off the circumflex branch of the left coronary artery in 10% of the population and in those with left dominance [22].

After its origin from the RCA, it merges into the cardiac wall towards the right fibrous trigone, lying in the vicinity of the mitral valvular fibrous ring [21]. It then terminates in the area of the apex of the triangle of Koch, where it supplies the AV node and adjacent structures [21, 22]. The fact that the distal end of the AV nodal artery terminates at the apex of the triangle of Koch makes it a reliable landmark for angiographic localisation of the AV node within the triangle of Koch [23]. This anatomic landmark also serves as a useful landmark to predict the possibility of an AV block during catheter ablation of AV nodal re-entrant tachycardia (AVNRT) [23].

The artery to the AV node is at risk of damage during mitral, aortic, and tricuspid valve surgery [23]. The close relationship of the AV nodal artery to the mitral valve attachment, especially at the area of the left proximal part of the posterior leaflet, makes it susceptible to intraoperative damage during manipulation of the mitral valve annulus fibrosus [24]. Injury to this artery will result in AV nodal injury, leading to rhythm disorders [22].

In patients with ostium primum defects, the artery to the AV node lies immediately beneath the inferior (dorso-caudad) rim of the defect, making it susceptible to injury during surgery [25]. This is unlike the septum secundum defect, which lies away and superior to the artery to the AV node, and the atrioventricular canal defect, which lies anterior to the artery to the AV node [25].

### 3.5 Acute or Right Marginal Artery

The acute marginal branch or right marginal artery usually originates at least 1 cm proximal to the acute margin of the RV before coursing towards the apex of the heart to supply the lateral wall of the RV [5, 26]. Although it is one of the largest branches of the RCA, it is not a typically preferred target for coronary artery bypass grafting [27]. However, this vessel can be a good bypass graft target [27, 28]. Resolution of a complete heart block has also been noted after revascularization of a blocked acute marginal branch of the RCA. This is believed to have been due to the elicited Bezold-Jarisch reflex from the increased vagal tone reflex, which was subsequently cut off by revascularization. This mechanism is unrelated to the arterial supply to the AV node [29].

### 3.6 Kugel’s Artery

Kugel’s artery is formed by the anastomotic connection between the branches of the distal RCA and the proximal circumflex artery [30]. It may also arise from the proximal RCA and terminate in the distal segment of the RCA, from the proximal circumflex artery to the RCA or even from the circumflex artery to the distal segment of the same artery [30-32].

The clinical importance of this artery is that in coronary artery disease, it may maintain myocardial viability in the region of RCA *via* a steal phenomenon to supply blood to affected epicardial vessels [30] or by providing collaterals through AV nodal artery and other septal collaterals [32].

### 3.7 Posterior Descending Artery (PDA)

The posterior descending artery (PDA) is one of the terminal branches of the RCA. It courses along the inferior interventricular groove to give rise to inferior septal perforators. It supplies the posterior wall of the right ventricle, the posterior one-third of the interventricular septum, and the diaphragmatic and posterior surface of the left ventricle in most cases [26].

The branches of the PDA that supply the posterior one-third of the interventricular septum are called the septal perforators.

### 3.8 Posterior Left Ventricular Artery (PLV)

The posterior left ventricular artery (PLV) is one of the terminal branches of the RCA and supplies the posterior/inferior wall of the left ventricle.

### 3.9 Injury to the RCA During Surgery and Ablation of Arrhythmias

Injury or occlusion of the RCA can occur rarely in patients undergoing tricuspid annuloplasty, and this has been reported during both De Vega annuloplasty and ring annuloplasty [33]. The risk of this injury may be higher in those with massive dilatation of the mitral valve annulus [33].

The site of injury or occlusion of the RCA during tricuspid annuloplasty is the area between the right marginal artery and the cardiac crux [33]. This corresponds to the area between the anteroposterior commissure (AP) and posterior leaflet of the tricuspid valve along the right ventricular free wall [33, 34]. Bennani *et al.* described this area using a clock-face model to represent the tricuspid annulus. With the anteroposterior commissure placed at the 12:00 position, the zone of high proximity between the RCA and the tricuspid annulus lies between 1:30 and 3:00 with a minimal distance of 4mm at 2:00 [35]. At this point, the right coronary artery is very close to the tricuspid annulus [33, 34]. It is hence advisable to place more superficial stitches in the area between the AP commissure and posterior leaflet annulus of the tricuspid annulus during annuloplasty to prevent injury to RCA [35]. However, if occlusion or injury inadvertently occurs, a percutaneous approach or CABG with vein grafting can be used for coronary revascularization [36].

The RCA can also be injured during ablation of arrhythmias. Damage can occur during posteroseptal pathway ablation and cavotricuspid isthmus ablation [37, 38]. The posterolateral branch (posterior left ventricular branch) of the RCA has been reported to be the most frequently injured vessel during posterolateral septal ablation. Studies have shown that the RCA is more separated from the cavotricuspid isthmus on the septal aspect but has a close relationship on the lateral aspect [39, 40].

The marginal artery, RCA, and PDA can be kinked, obstructed, or injured during internal plication of the atrialized right ventricle in the Cone procedure for patients with Ebstein anomaly. To avoid this injury, suture bites should be confined to the endocardium and should not exit onto the epicardial surface [41].

### 3.10 Myocardial Ischaemia Involving RCA

Posterior descending artery (PDA) is preferred for revascularization when the RCA is the target for CABG. Patency rates have been shown to be better distal to the crux, even if an appropriate segment of RCA is amenable proximal to the crux [42]. This is believed to be due to the accelerating effect of low shear stress on atherosclerosis, which is more prominent at sites of bifurcation [42]. This may cause venous grafts anastomosed proximal to the crux to be occluded earlier than those anastomosed distal to the crux [42]. The anastomosis performed distal to the crux would also not be exposed to increased vascular resistance and would be able to maintain its flow with an adequate distal run-off [42].

In inferior myocardial infarction resulting from RCA occlusion, ischaemic mitral regurgitation may occur due to posteromedial papillary muscle rupture [43]. This is because the posteromedial papillary muscle is only supplied by the PDA [43, 44]. Conversely, rupture of anterolateral papillary muscle rupture is relatively less common as it has a dual blood supply from the first diagonal branch of the LAD and the first obtuse marginal (OM) branch from the circumflex artery [43, 44].

### 3.11 Congenital Abnormalities Involving RCA and its Clinical Importance

**Fig. (2):**
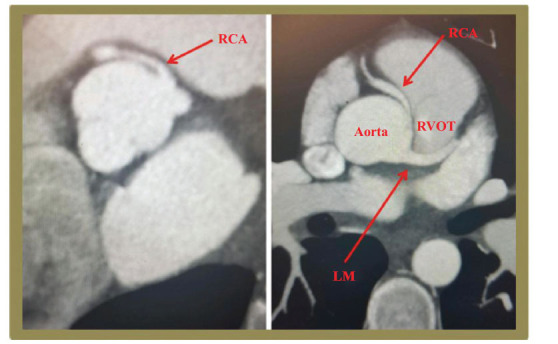
CT Angiogram showing the right coronary artery (RCA) originating from the left coronary cusp adjacent to the left main coronary artery (LMCA). Reproduced with kind permission from Prof Bassam Omar: Kolakalapudi P, Sachdev S, Omar B. Anomalous Coronary! Is It Time To Panic! Cardiofel Newslet 2019; 2: 13-14.

**Fig. (3):**
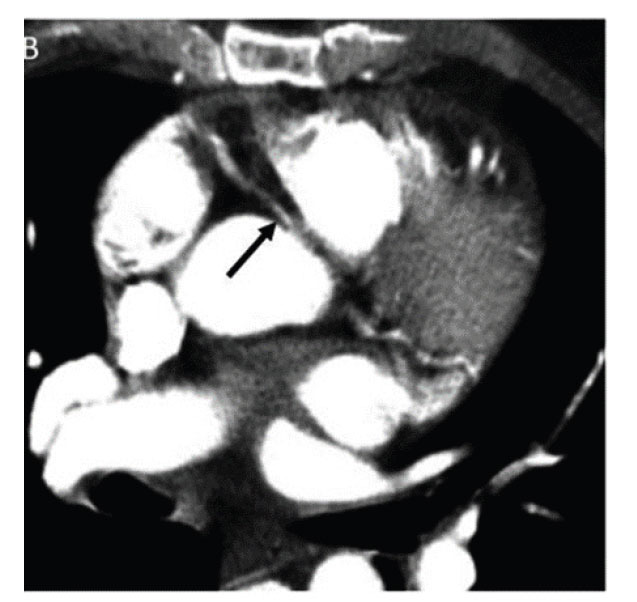
Axial contrasted CT scan of the chest showing an anomalous RCA arising from the left sinus of Valsalva coursing between the aorta and pulmonary artery with the proximal RCA with an oval/slit-like appearance suggesting an intramural course. Reproduced with kind permission from Dr Clay: Clay KJ, Hamid A, Deere BP. Two Cases of Highly Symptomatic Interarterial Anomalous Right Coronary Arteries. Cureus. 2023; 15: e42761.

Anomalous origin of the RCA from the left aortic sinus of Valsalva has been reported (Fig. **2**) [45-47]. In cases with a high interarterial course between the aorta and pulmonary artery, there is a risk of compression of the vessel, which could result in angina, malignant arrhythmias, and sudden cardiac death [46, 47]. The anomalous origin may have a slit-like orifice, an acute angle of origin, and intramural course (Fig. **3**).

There have been reports of intraatrial RCA courses, and these tend to occur in the middle and late stages of the disease [48]. This predisposes the RCA to a high risk of injury during surgical procedures involving the right atrium and difficulty localizing distal RCA if planned preoperatively as a target for grafting [48, 49]. It is also at risk during interventional procedures like pacemaker insertion and ablation [48, 49].

In shepherd crook deformity of the RCA, the ostium is oriented superiorly, and the proximal RCA courses upwards before making a U-turn to enter the right atrioventricular groove. It poses a significant challenge during percutaneous transluminal angioplasty [50].

RCA may become duplicated and present with the same or separate ostia in the aortic sinus [51]. It has important implications for ostial cardioplegic delivery during aortic valve surgery and while performing procedures that require translocation of RCA as with aortic root procedures, including Ross procedure, Nikaidoh procedure and modified Bentall procedure [51].

In high take-off RCA, the coronary ostium is 1cm or more above the sinotubular junction [52]. This condition is more common in patients with bicuspid aortic valve [4, 53]. There is a high risk of sudden death, especially in those with intramural, interarterial or acute angle take-off [52]. The surgical importance includes the risk of unexpected injury during aortic surgery, unsuccessful induction of cardioplegia if an aortic clamp is applied below a high take-off RCA, and technical challenges in performing coronary angiograms [4, 52, 54, 55].

## 4 APPLIED ANATOMY OF THE LEFT MAIN CORONARY ARTERY (LMCA)

**Fig. (4):**
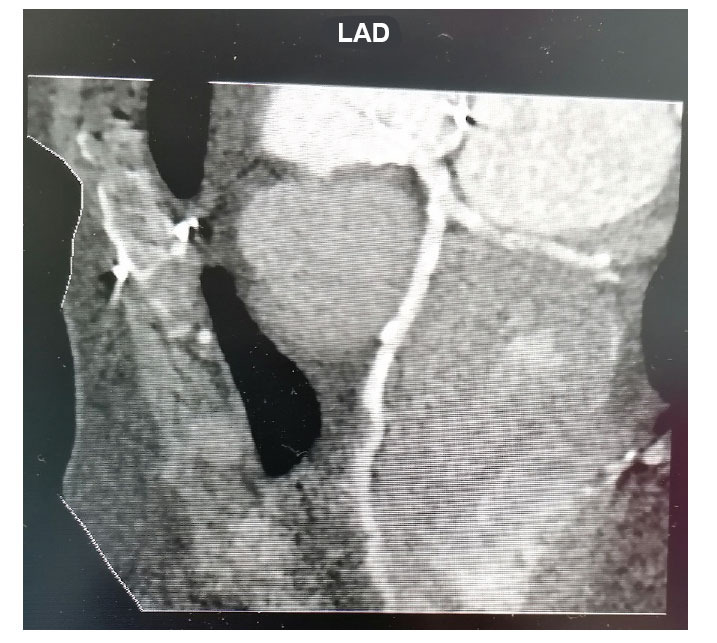
LMCA dividing into LAD and Circumflex artery.

The left main coronary artery (LMCA) originates from the left sinus of Valsalva, passes behind the pulmonary trunk and gives rise to the left anterior descending artery (LAD) and the circumflex artery (Fig. **4**). Sometimes, it trifurcates 25% of individuals to give origin to an intermediate branch, often called ramus intermedius [56]. When present, the ramus intermedius takes the normal course of a high obtuse marginal branch of the circumflex artery.

LMCA has a mean diameter of 5 mm (3.5 mm – 6.5 mm) and the length ranges between 10.5 mm ± 5.5 mm [57, 58].

The artery can be divided into the ostium, the trunk and the bifurcation [59]. The ostium of the left main has a higher proportion of smooth muscle and elastic tissue than the rest of the coronary vasculature [60].

### 4.1 Length of LMCA and Delivery of Ostial Cardioplegia

In cardiac surgery patients with short LMCA requiring ostial cardioplegia, the tip of the cannula may extend beyond the bifurcation of the left main stem and selectively intubate and perfuse either the LAD on most occasions or the circumflex artery [61, 62]. This results in inadequate intraoperative myocardial protection. Erasmi *et al.* reported a sign that suggests selective intubation of only one branch of the left main trunk in short LMCA [61]. These patients observed a retrograde return of desaturated blood from the nonperfused branch out of the ostium of the left main stem around the cannula [61]. This is unlike in most patients where selective intubation of the left main trunk ideally leads to backflow of desaturated blood from the right coronary ostium [61, 62].

Patients with bicuspid aortic valves have been shown to have a higher incidence of short LMCA and left coronary artery dominance [63].

The ostium of the LMCA may develop stenosis after aortic valve replacement (AVR) [64, 65]. The incidence of stenosis involving coronary ostia following AVR is 1-5%, and stenosis can be acute or delayed [64, 65].

### 4.2 Congenital Abnormalities Involving LMCA and its Clinical Importance

**Fig. (5):**
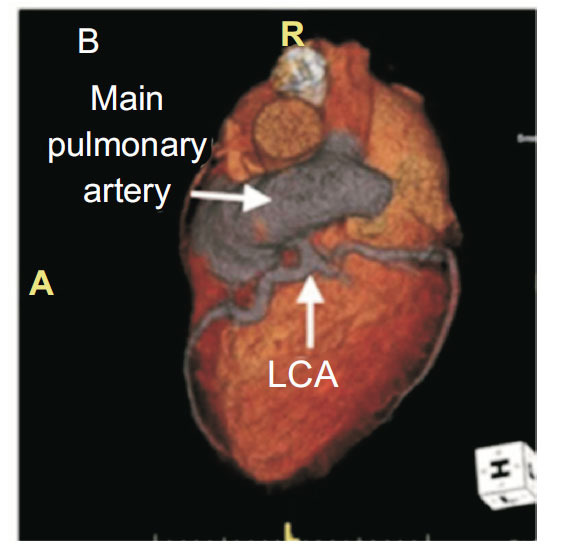
Volume-rendered (VR) 3D images showing the anomalous origin of a dilated left coronary artery (LCA) from the main pulmonary artery (MPA). Reproduced with kind permission from Dr Pedro Teixeira: Teixeira P, Silva M. Aborted Sudden Cardiac Death in a Young Adult: An Exceptionally Rare Cause. Cureus 2020; 12(10): e11013. doi:10.7759/cureus.11013.

Origin of the LMCA from the pulmonary artery can rarely occur and is referred to as Anomalous origin of Left Coronary Artery from the Pulmonary Artery (ALCAPA) or Bland-Garland-White syndrome (Fig. **5**) [66-68]. Infantile variety may present with congestive heart failure and myocardial infarction, while adult‐type ALCAPA are most commonly asymptomatic or have subclinical ischemia and may survive till adulthood [67, 68].

The LMCA may originate from the right sinus of Valsalva. It may potentially take four (4) different courses when this happens [69]. It may pass between the aorta and pulmonary trunk, posteriorly and adjacent to the pulmonary trunk, or pass anteriorly over the right ventricular outflow tract (RVOT). The LMCA may also course along the crista supraventricularis intramyocardially or subendocardially, surfacing in the proximal interventricular sulcus or may rise to the right of the RCA and pass posteriorly to the aortic root [69]. The clinical significance of this anomalous origin is the possibility of compression between structures resulting in myocardial ischaemia or sudden death [69, 70].

High take-off of LMCA has also been reported [71, 72]. It occurs less commonly than high-take-off of the RCA [71]. As in high take-off RCA, it runs a risk of iatrogenic injuries during cardiovascular interventions and can predispose to inadequate myocardial protection during cardiac surgery. It may pose significant challenges during aortotomy for aortic valve replacement, major aortic root reconstruction and supracommissural replacement of ascending aorta [71, 72].

The abnormal origin of LMCA and RCA is common in truncus arteriosus. This is particularly important in the primary repair and later redo of the truncal valve. Lenox *et al.* described a high incidence of coronary ostial and arterial abnormalities in Truncus arteriosus. These include left coronary ostium in a posterior and high position, stenosis of the coronary ostium caused by small size, slit-like shape, or the location of the ostium above or in a commissure and the acute angle take-off of the coronary artery [73].

LMCA is potentially vulnerable in the Ross procedure when harvesting autograft. It is also vulnerable in redo Right Ventricular to Pulmonary Artery (RV-PA) conduit replacement, where the homograft conduit may be densely adherent to the LMCA.

### 4.3 Applied Anatomy of the Left Anterior Descending Artery (LAD)

This artery is one of the two branches of the LMCA, almost arising as its continuation [74]. It emerges on the anterior surface of the heart between the root of the pulmonary trunk and the left auricular appendage and subsequently runs along the anterior interventricular septum towards the apex of the heart [75].

**Fig. (6):**
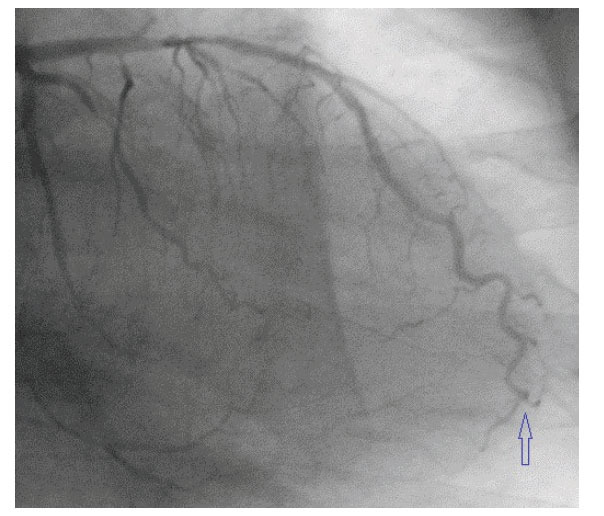
Moustache sign in LAD in angiogram.

Angiographically, it is recognized by the course of the vessel towards the apex of the heart, the septal perforators which exit from the vessel at the right angle, and its bifurcation at the apex akin to a mustache or the bifid tail of a whale. This has been described as a “whale tail sign”, “pitchfork sign”, or “Moustache sign” (Fig. **6**) [76].

**Fig. (7):**
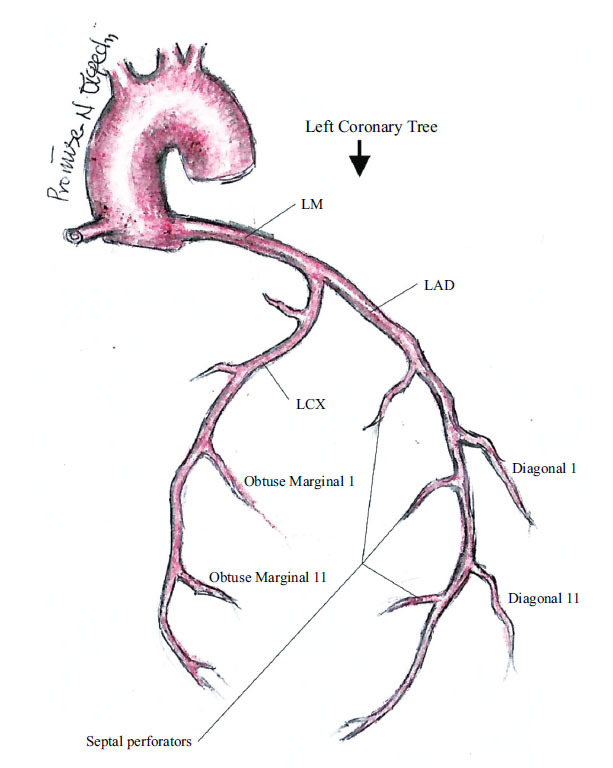
Branches of LAD and Circumflex artery.

It gives off the diagonal branches and the septal perforators in the AV sulcus between the left and right ventricle (Fig. **7**).

### 4.4 The LAD is Divided into 3 Parts

1. Proximal one-third: This extends from the origin of the LAD to the origin of the first septal perforator.
2. Middle one-third: This extends from the first septal perforator to the origin of the last diagonal artery.
3. Distal one-third: This extends from the last diagonal branch to the termination of the LAD.

### 4.5 Diagonal Branches

The diagonal branches arise at an acute angle and run diagonally on the anterolateral portion of the left ventricle, supplying the anterolateral wall of the left ventricle [77]. They are designated D1 (first diagonal), D2 (second diagonal) *etc*.

### 4.6 Septal Perforators

The septal perforators, averaging about 9 in number (range 6-14), arise at right angles from the LAD and penetrate the interventricular septum to supply the anterior two-thirds of the muscular ventricular septum [78, 79].

The first septal perforator is more pronounced. It resides within the muscular (moderator) band in the upper part of the interventricular septum and surfaces 1cm below the nadir of the left-facing cusp [78, 80]. It then reaches the anterior papillary muscle of the tricuspid valve. This muscle therefore, serves as a landmark to locate the first (main) septal perforating artery.

The first septal perforator is at risk of injury during the Ross procedure during autograft harvest [81]. Precautions taken by the surgeon during this procedure include transecting the muscle in a horizontal plane/enucleating to ensure that only the right ventricular portion of the interventricular septal muscle is cut and avoiding deep dissection into the septum where the artery can be damaged [78, 80]. During proximal anastomosis of the pulmonary homograft, partial thickness bites should be taken posteriorly through the interventricular septum [80].

The septal perforating branch of the LAD supplies the moderator band, which houses the right bundle branch. Thrombosis of this artery can lead to right bundle branch block, complete heart block, and infarction of the anterior interventricular septum [78, 82].

Knowledge of the applied anatomy of septal perforators is important in alcohol septal ablation, one of the well-established modalities of treatment of hypertrophic obstructive cardiomyopathy [83]. This procedure involves the injection of 1 to 4 ml of 96% ethanol into a septal perforator branch of LAD [83]. This results in infarction of the basal septum, septal scarring, and thinning, resulting in the widening of the LV outflow tract [83].

There exist important collaterals or anastomoses between the proximal and distal anterior septal perforating branches of the LAD and between the septal perforating branches of the LAD and the PDA [78, 84, 85]. These collaterals serve to maintain some degree of myocardial viability in cases of significant obstruction either in the RCA or left coronary artery [78, 84-86].

### 4.7 Right Ventricular Branches

Branches supplying the right ventricle also arise from the LAD.

### 4.8 Myocardial Bridging

Myocardial bridging is a terminology used to describe a coronary artery that dips intramyocardially along its course instead of the normal epicardial course [87]. Myocardial bridge is used to describe the muscle overlying the artery, while the intramyocardial segment is referred to as a tunneled artery [87]. The most common vessel implicated in the LAD in 67-98% of myocardial bridging, with the proximal and mid-segment being the most commonly involved [87, 88]. Most patients with myocardial bridging are asymptomatic, as coronary blood flow occurs primarily during diastole. However, ischaemic symptoms, myocardial infarction, and even sudden cardiac death have all been reported [89-91]. The diagnosis is often made by coronary angiography, which shows a significant reduction in vascular lumen during systole due to compression. Other investigative modalities include intravascular ultrasound (IVUS) or optical coherence tomography (OCT) [92]. Access to a tunneled artery can be challenging during coronary artery bypass grafting (CABG).

### 4.9 LAD and Coronary Artery Disease

The gold standard of CABG is the left internal mammary artery (LIMA) to LAD anastomosis because of its excellent long-term patency rate.

Myocardial infarction involving significant areas of the anterior, septal and apical portions of the myocardium takes place in LAD occlusion. Acute anterior infarcts typically result in apical ischaemic ventricular septal defect (VSD), while the inferior or basal infarction will likely cause basal defects at the base of the junction between the septum and the posterior wall [93]. Apical defects occur at the same level on both sides of the septum and are usually small, have thin margins, and are more likely surrounded entirely by septum [93]. This is unlike the inferior infarcts that more commonly result in complex ruptures with a complex course through a haemorrhagic and necrotic basal inferoposterior septum and also may be associated with intramyocardial dissection and involvement of the free wall [93-95].

In redo sternotomy in patients with patent LIMA-LAD graft, there is a risk of intraoperative injury to graft, and finding a coronary artery target for the graft may be difficult [96, 97].

### 4.10 Congenital Abnormalities Involving LAD and the Clinical Relevance

**Fig. (8):**
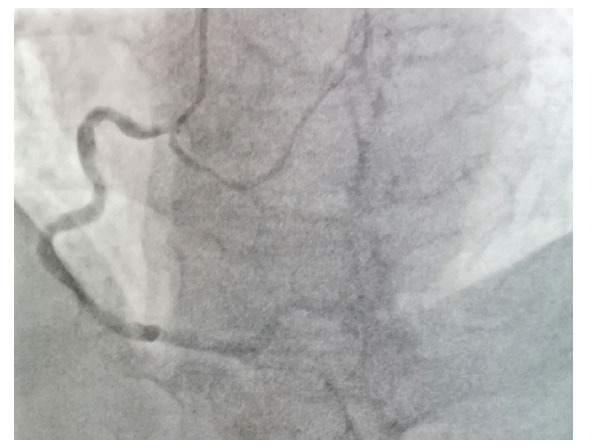
Angiogram showing anomalous origin of LAD from right aortic sinus.

Several congenital malformations of important clinical significance involving the LAD have been described [69, 98]. The LAD and the circumflex artery may originate separately from separate ostia in the left sinus of Valsalva. Failure to recognize this may cause confusion during coronary angiography [98]. LAD may also arise from the RCA, right sinus of Valsalva (Fig. **8**), and even rarely from the pulmonary artery (ALCAPA) [98-101]. These anomalies may be associated with an intramural inter-arterial course, in which case there is an increased risk of myocardial ischaemia and sudden cardiac death [98-101].

Successful percutaneous coronary intervention (PCI) has recently been reported in patients with an Anomalous coronary artery originating from the opposite sinus of Valsalva (ACAOS) [102-104]. The combination of an intramural course, a slit-like ostium, an acute take-off, and an abnormal shape of the vessel mimics a complex stenosis that requires surgical deroofing [102]. However, in patients with significant comorbidities, PCI using an intravascular ultrasound scan (IVUS) has been reported to reduce symptoms [105]. Stent angioplasty has been shown to increase the cross-sectional area of the intramural segment, eliminate pulsatility of the segment, and eliminate lateral compression [105]. In-stent restenosis varied between 9-40% [102, 105]. Revascularization with balloon angioplasty and drug-eluting stent has also been reported in a patient with an anomalous left main artery originating from the right coronary sinus who presented with inferior myocardial infarction [106].

**Fig. (9):**
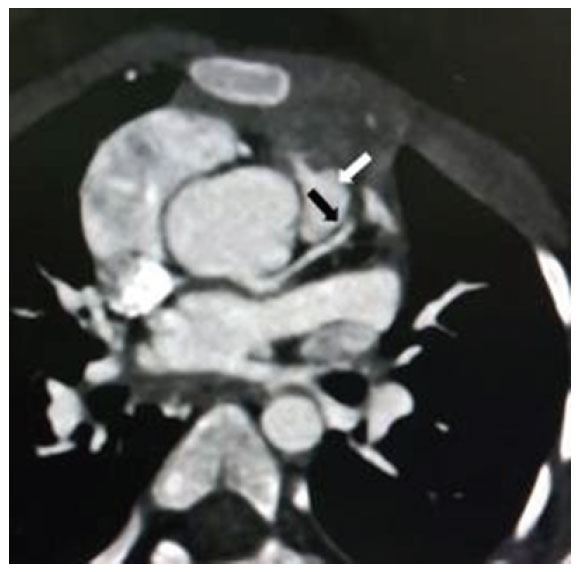
LAD passing close to pulmonic valve. Reproduced with kind permission from Prof T Ashraf:Ashraf T, Farooq F, Syed Muhammad A, Akhtar P, Khan M, Khuwaja AM, Khan MN, Karim M. Coronary Artery Anomalies in Tetralogy of Fallot Patients Undergoing CT Angiography at a Tertiary Care Hospital. Cureus 2020; 12: e10723.

About 3-5% of patients with TOF may have an anomalous LAD originating from the RCA or right sinus of Valsalva and coursing anteriorly over the RVOT (Fig. **9**) [107, 108]. The vessel will be at risk of injury resulting in myocardial ischaemia during repair with the transannular patch or RVOT patch. In these patients, some congenital cardiac surgeons may opt for temporary palliative measures instead of a single-stage complete repair to allow for somatic growth [109], while others will proceed with transarterial repair without ventriculotomy and in those with very small RVOT and right ventricular hypertension, a right ventricular to pulmonary artery (RV-PA) conduit may be opted for to preserve the course of the vessel [110]. There is a 10.3% combined risk of encountering an anomalous coronary artery (usually LAD) or a large conus artery crossing the RVOT in these patients [111, 112].

Duplication or dual LAD has also been reported [113, 114]. In these patients, two LADs are identified as a short LAD and a long LAD. Four types have been described. [115]. The ‘short’ and ‘long’ LAD arise from the LMCA in Types 1-3, whereas in Type 4 which is less common, the ‘short LAD’ arises from the LMCA while the ‘long LAD’ arises from the RCA [113-116]. The clinical importance of this anomaly is the potential confusion that may arise in the interpretation of angiography and in the planning of CABG [116-119]. Dual LAD may occur in association with TOF and complete transposition [119].

Hyper-dominant or super-dominant LAD is a rare coronary anomaly in which LAD gives rise to PDA instead of left circumflex (left dominance) or right coronary artery (right dominance) [120, 121]. The clinical implication of this anomaly is that the hyper-dominant LAD supplies a larger area of the myocardium than in left and right dominance. Hence, if it is occluded, an infarction of the anterior wall, septum, and inferior wall will occur, and the patient may present with cardiogenic shock [120]. Varga *et al.* described an extreme level of LAD-dominant circulation, with the LAD not only giving rise to the PDA but making a 180° turn at the crux and continuing apically as the posterolateral artery [121].

**Fig. (10):**
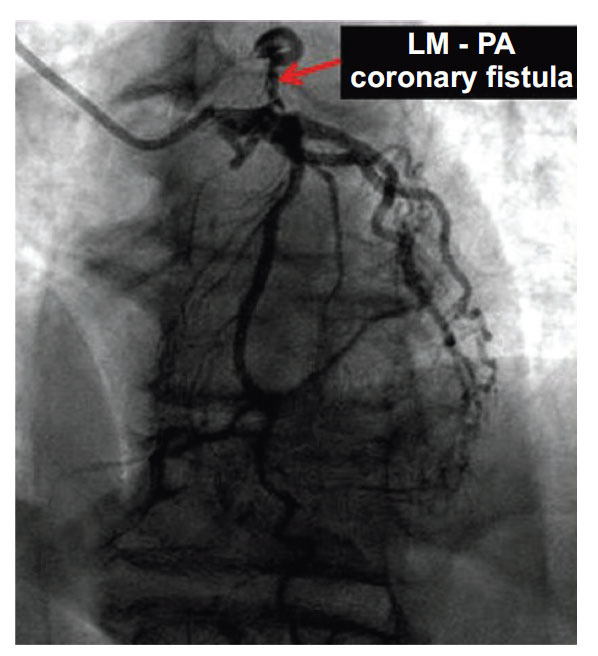
Coronary angiogram showing LMCA to PA fistula. Reproduced with kind permission from Prof Bassam Omar: Pierluissi Rivera V, Omar B. Left Main to Pulmonary Artery Coronary Fistula: Surgical Closure! Cardiofel Newslet 2022; 5: 8-11.

Coronary artery fistula involves the termination of the coronary artery or its branches into the cardiac chamber or low-pressure vascular structure like the pulmonary artery (Fig. **10**). It should be distinguished from systemic arterial termination as they are dilated and tortuous compared with other branches [4]. In coronary fistula, the more proximal the feeding artery originates from the main coronary artery, the more dilated it tends to become [122, 123]. The consequence of the presence of a fistula depends on the fistula size and flow pattern [122, 123]. Those terminating in the right-sided chambers or pulmonary arteries and producing significant left to right shunts may cause pulmonary hypertension or biventricular volume overload, while those terminating in the left atrium or left ventricular fistulae may produce left ventricular volume overload, myocardial hypertrophy as well as myocardial ischaemia from coronary artery steal resulting in angina [122, 123].

## 5 APPLIED ANATOMY OF THE CIRCUMFLEX ARTERY

The circumflex artery is embedded in fatty tissue at its origin from the LMCA beneath the left atrial appendage. It enters the left atrioventricular groove and terminates near the obtuse margin of the left ventricle [75]. In individuals with left dominant coronary circulation, it extends to the cardiac crux, giving off the artery to the AV node and beyond the cardiac crux, giving off the PDA, which supplies the posterior portion of the interventricular septum [5, 74].

### 5.1 Obtuse Marginal Branches

In its course in the left AV groove, it gives off 2-3 obtuse marginal (OM) branches that supply the lateral free wall of the left ventricle, branches to the left atrium, and a branch to the sinoatrial node in 40-50% of cases [5, 74, 75].

### 5.2 Circumflex Artery and CABG

In coronary artery bypass grafting, obtuse marginal (OM) branches of the circumflex artery are targeted to bypass circumflex artery stenosis. Bypass grafting is most easily performed to the OM branches other than to the circumflex artery itself because of difficulties in exposing the circumflex artery in the atrioventricular groove, where it lies deep into the coronary sinus [124, 125].

### 5.3 Injury to the Circumflex Coronary Artery During Surgeries

Injury to the circumflex coronary artery may result during mitral valve surgeries. Incidence of circumflex artery injury after mitral valve operation ranges from 0.5-1.8%. However, in high-volume centres, it can be as low as 0.15% [64, 126]. Husain *et al*. reported a 30-day mortality of 33.3% [122]. A high index of suspicion is required to diagnose this injury [127].

Injury to the circumflex artery is due to the anatomical relationship of this vessel to the posterior portion of the mitral valve annulus. The risk of injury to the circumflex artery has been reported to be higher in left dominant circulation and codominant circulation than in right dominant circulation [128-130]. The distance between the circumflex artery and the mitral valve annulus at the level of the anterior commissure in left dominant circulation has been measured to be 1mm compared to an average of 4.5 mm in codominant circulation and 8mm in right dominant circulation [128]. For the same reason, the proximal one-third of the vessel is at the greatest risk during valve surgery [131]. If the vessel is incidentally injured during a mitral valve procedure, emergency CABG with saphenous vein or suture correction can reduce the possibility of serious myocardial necrosis if the occlusion is recognized intraoperatively, while percutaneous coronary intervention (PCI) may be appropriate when it is recognized in the immediate postoperative period if the vessel is distorted [131, 132].

The circumflex artery and the LMCA, to a lesser extent, are also at risk of occlusion or distortion during the application of left atrial appendage occlusion device during open surgery and thoracoscopic procedure [133-135].

### 5.4 Congenital Anomalies Involving the Circumflex Artery

Anomalies of the circumflex artery may include the absence of the artery or anomalies of its origin [136-138]. Among the anomalous origin of this artery, origin from the right coronary sinus is the most common variant. [137, 138]. It usually occurs retroaortic and may involve separate ostia for RCA and circumflex artery (type 1), common ostia for both in the right sinus (type II), and circumflex artery originating as a proximal branch of RCA [137, 138]. Although the anomalous origin of circumflex from the right sinus of Valsalva is usually asymptomatic, it may pose a significant challenge in catheterization and a significant surgical challenge during emergency surgery for aortic root dissection [138, 139].

Acute take-off of the circumflex artery may pose a challenge during percutaneous coronary intervention [140].

## CONCLUSION

A good knowledge of the applied anatomy of the coronary artery is important to cardiac surgeons, interventional cardiologists and cardiac trainees. This review article has extensively discussed the anatomy of the coronary artery and common anomalies with emphasis on the clinical applications in adult and paediatric cardiac surgery and also for interventional cardiology.

## LIST OF ABBREVIATIONS

ALCAPA: Anomalous Origin of Left Coronary Artery from Pulmonary Artery
ASD: Atrial Septal Defect
AV Node: Atrioventricular Node
AVNRT: Atrioventricular Re-Entrant Tachycardia
AVR: Aortic Valve Replacement
CABG: Coronary Artery Bypass Grafting
IVU: Intravascular Ultrasound
LIMA-LAD: Left Internal Mammary-Left Anterior Descending Artery
LMCA: Left Main Coronary Artery
OCT: Optical Coherence Tomography
OM: Obtuse Marginal
PAPVC: Partial Anomalous Pulmonary Venous Connection
PDA: Posterior Descending Artery
PLV: Posterior Left Ventricular Artery
RCA: Right Coronary Artery
RV-PA Conduit: Right Ventricular to Pulmonary Artery Conduit
RVOT: Right Ventricular Outflow Tract
SA Node: Sinoatrial Node
SVC: Superior Vena Cava
VSD: Ventricular Septal Defect
